# The Evolution of Pulmonary Hypertension and Its Prognostic Implications Post-TAVI—Single Center Experience

**DOI:** 10.3390/medicina58091182

**Published:** 2022-08-30

**Authors:** Luiza Cristina Dumitrof, Igor Nedelciuc, Mihai Roca, Radu Crișan-Dabija, Traian Mihăescu, Grigore Tinică

**Affiliations:** 1 Department of Medical Specialties (I and III) or Surgical Specialties (I), University of Medicine and Pharmacy “Grigore T. Popa”, University Street nr 16, 700115 Iaşi, Romania; 2 Institute of Cardiovascular Diseases “Prof. Dr. George I.M. Georgescu”, 700503 Iași, Romania; 3 Clinical Rehabilitation Hospital, Cardiovascular Rehabilitation Clinic, Pantelimon Halipa Street nr 14, 700661 Iaşi, Romania; 4 Pulmonology Department, Clinic of Pulmonary Diseases, 700115 Iasi, Romania

**Keywords:** transcatheter aortic valve implantation, pulmonary hypertension, aortic stenosis, heart failure

## Abstract

*Background and Objectives*: Since the first transcatheter aortic valve implantation (TAVI) procedure was performed in 2002, advances in technology and refinement of the method have led to its widespread use in patients with severe aortic stenosis (AS) and high surgical risk. We aim to identify the impact of TAVI on the clinical and functional status of patients with severe AS at the one-month follow-up and to identify potential predictors associated with the evolution of pulmonary hypertension (PH) in this category of patients. *Materials and Methods*: We conducted a prospective study which included 86 patients diagnosed with severe AS undergoing TAVI treatment. We analyzed demographics, clinical and echocardiographic parameters associated with AS and PH both at enrolment and at the 30-day follow-up. *Results*: In our study, the decrease of EUROSCORE II score (*p* < 0.001), improvement of angina (*p* < 0.001) and fatigue (*p* < 0.001) as clinical benefits as well as a reduction in NYHA functional class in patients with heart failure (*p* < 0.001) are prognostic predictors with statistical value. Regression of left ventricular hypertrophy (*p* = 0.001), increase in the left ventricle ejection fraction (*p* = 0.007) and improvement of diastolic dysfunction (*p* < 0.001) are echocardiographic parameters with a prognostic role in patients with severe AS undergoing TAVI. The pulmonary artery acceleration time (PAAT) (*p* < 0.001), tricuspid annular plane systolic excursion (TAPSE) (*p* = 0.020), pulmonary arterial systolic pressure (PASP) (*p* < 0.001) and the TAPSE/PASP ratio (*p* < 0.001) are statistically significant echocardiographic parameters in our study that assess both PH and its associated prognosis in patients undergoing TAVI. *Conclusions*: PAAT, TAPSE, PASP and the TAPSE/PASP ratio are independent predictors that allow the assessment of PH and its prognostic implications post-TAVI.

## 1. Introduction

Aortic stenosis (AS) is the most common valve disease worldwide, affecting mainly the elderly over 60 years [1]. It has a prevalence directly proportional to patient age and a clinical picture dominated by a slow progression of subclinical symptoms over several decades [2]. The morbidity and mortality rate increases sharply with worsening clinical signs and symptoms, requiring prompt therapeutic management [3]. The evolution over time of percutaneous intervention techniques has led to the extension of the indications for interventional treatment of valvulopathies as an alternative to classical surgical treatment [4,5]. 

Surgical aortic valve replacement (SAVR) was for many decades the only method of treatment for these patients [6]. Transcatheter aortic valve implantation (TAVI) is recommended in patients with moderate to severe perioperative risk assessed using risk scores as well as in the case of low-risk patients over 75 years old [7,8]. Clinical studies in the literature give this minimally invasive treatment method a superior SAVR prognosis in the short and medium term regardless of risk category [9,10,11,12]. The management of TAVI patients is complex and multidisciplinary. In addition to the correction of valvulopathy by TAVI, drug treatment of associated heart failure and cardiovascular recovery play an important role as integrative elements that contribute to improving both short- and long-term prognosis [13,14]. In patients undergoing valve replacement surgery (TAVI or SAVR), cardiac rehabilitation facilitates the restoration of functional status and increases the patient’s quality of life, with both short- and long-term prognostic benefits [15,16].

The presence of pulmonary hypertension (PH) in patients with severe AS is an indirect indication of exhaustion of the compensatory mechanisms of the heart and therefore increased mortality in the absence of prompt therapeutic management [17]. PH (presence of a pulmonary artery systolic pressure above 40 mmHg) is associated with an increased morbidity-mortality rate at 1 year despite TAVI due to progression of signs and symptoms of heart failure [18,19,20]. Recent studies attest to the non-inferiority of TAVI to SAVR in patients with severe AS and low surgical risk, the main concerns of the scientific community continue to be the assessment of long-term prognosis and the impact of long-term residual aortic regurgitation [11,21]. From 2002 to the present, the refinement of the method and the devices used has resulted in the use of seven distinct new generation prostheses with reduced size and minimal regurgitation [22,23].

The aim of this study was to identify the impact of TAVI on the clinical and functional status of patients with severe AS at the one-month follow-up and to identify prognostic factors associated with the evolution of PH in this category of patients.

## 2. Materials and Methods

### 2.1. Study Design

We conducted a prospective study which included 86 patients diagnosed with severe AS undergoing interventional TAVI treatment. The diagnosis of aortic valvulopathy was established according to the European ESC guideline for valvular heart disease [16]. The indication for surgical correction has been established in patients with severe AS: aortic peak velocity above 4 m/s, with a mean gradient above 40 mmHg and an aortic valve area of less than 1 cm^2^. The EUROSCORE II score was calculated for all patients to assess the risk of morbidity and mortality [24]. 

TAVI was performed in elderly AS patients (over 65 years old), with a EUROSCORE II value of over 4%, frail, with various associated aortic pathologies (porcelain aorta or previous thoracic irradiation) and with a life expectancy of less than 10 years, but more than one year which associates with a procedure success rate of over 25% for a minimum of 2 years. All 86 patients underwent multimodal imaging evaluation by echocardiography and cardiac computed tomography to assess functional and structural parameters and to further identify risk factors associated with improved prognosis. The data provided by the two investigations facilitated the diagnosis as well as the assessment of treatment-related features such as the size of the prosthesis or changes in the local anatomy. After TAVI, all patients were monitored in the intensive care unit. 

Patients who underwent SAVR or those with contraindications to TAVI (such as active endocarditis, thrombi in the ascending aorta, short distance between annulus and the coronary ostium or an annulus size smaller than 18 mm or bigger than 29 mm) were excluded from the study. Demographics, vital signs on admission, comorbidities or echocardiographic parameters were obtained from the observation charts.

Renal function assessment (creatinine and serum urea) was performed prior to TAVI, and electrocardiogram and vital parameters were monitored throughout the interventional procedure.

Tricuspid annular plane systolic excursion (TAPSE)/pulmonary arterial systolic pressure (PASP) ratio is an independent prognostic factor associated with hemodynamic status and functional class on the basis of which the severity of PH was assessed [25]. In our study, statistical analysis revealed a preoperative mean value of 0.45 for the TAPSE/PASP ratio and according to which two study subgroups were formed: group 1 with 45 patients (52.3%) with a ratio value less than 0.45 and group 2 with 41 patients (47.7%) with a ratio value above 0.45. None of the patients evaluated 30 days after TAVI died. 

### 2.2. Statistical Analysis

The descriptive analysis was performed using SPSS statistics software (version 26 for Windows; SPSS Inc., Chicago, IL, USA). Continuous variables were reported as the mean ± SD and categorical ones as a number (frequency and percentages). The Kolmogorov–Smirnov test was used to assess the normal distribution of the data. Continuous variables were compared using the *t*-test (parametric analysis). Categorical variables were compared using Fisher’s exact test. Groups were compared using the χ^2^ or Fisher’s exact test for categorical variables and ANOVA/Student’s *t*-test and Wilcoxon–Mann–Whitney U/Kruskal–Wallis tests for continuous variables, depending on their distribution.

A *p*-value of ≤0.05 was considered statistically significant. Receiver operating characteristic analyses were performed to calculate area under the curve for clinical parameters. 

### 2.3. Ethics

The study was approved by the Ethics Committee of the University of Medicine and Pharmacy “Grigore T. Popa” Iași and of the Cardiovascular Diseases Institute Iași, and was conducted according to the Helsinki Declaration. All patients signed an informed consent statement, which mentioned that the results would be used for research purposes. 

## 3. Results

We enrolled in our study 86 patients diagnosed with severe AS undergoing TAVI who were evaluated preoperatively and one month after the interventional procedure. Demographics, resting hemodynamics, echocardiographic findings at baseline and at 1-month follow-up are presented in Table 1. Predominantly female patients were enrolled in the study (53.3%), with an average age of 75.8 ± 7.44 years old. 

Three main types of aortic prostheses have been used for TAVI, most commonly Edwards Lifesciences Sapien 3 in 74.4% of cases. Regarding the vascular approach, in 83.7% of cases the transfemoral approach was chosen, the transapical approach being used in a small percentage of patients of only 16.3%. The EUROSCORE II score was assessed preoperatively with an mean value of 8.92 ± 10.62. 

At the one month follow-up, the percentage of patients with angina (73.3 vs. 34.9%) or fatigue (9.3 vs. 47.7%) had decreased significantly compared to enrolment. Atrial fibrillation was electrocardiographically assessed in a small percentage of patients both preoperatively and one month after TAVI (24.4 vs. 25.6%, *p* = 1). Heart failure is a statistically significant parameter in our study, with TAVI leading to improvement in functional status after one month (*p* < 0.001). The percentage of patients with symptoms specific to NYHA functional class III improved considerably at one month after TAVI (73.3 vs. 18.6%). No patient had symptoms suggestive of NYHA class IV at the one-month follow-up evaluation.

TAVI also resulted in improvement of hemodynamic parameters in both systolic (149.07 ± 21.17 mmHg vs. 134.15 ± 11.21 mmHg, *p* < 0.001) and diastolic blood pressure (82.72 ± 9.79 mmHg vs. 78.59 ± 8.64 mmHg, *p* < 0.001) as a result of drug treatment of hypertensive patients. Mean arterial oxygen saturation significantly improved one month after TAVI (89.76 ± 2.62% vs. 96.65 ± 1.59%, *p* <0.001). 

Echocardiographic parameters were also assessed both preoperatively and one month after TAVI. Thus, the maximum aortic gradient (95.42 ± 24.86 mmHg vs. 24.06 ± 13 mmHg, *p <* 0.001), the mean aortic gradient (59.22 ± 15.49 mmHg vs. 13.42 ± 9.83 mmHg, *p <* 0.001), aortic peak velocity (4.79 ± 0.59 m/s vs. 2.34 ± 0.51 m/s, *p* < 0.001) and aortic valve area (0.69 ± 0.34 vs. 2.51 ± 0.39 cm^2^, *p* < 0.001) improved significantly at one month, being statistically significant parameters in our study group. Assessment one month after TAVI also showed improvement in LV ejection fraction (54.45 ± 10.39% vs. 55.88 ± 9.45%, *p* = 0.007) and decrease in LV end-systolic diameter (34.85 ± 7.48 mm vs. 32.27 ± 7.17 mm, *p* = 0.019) and interventricular septum (14.42 ± 1.80 mm vs. 13.86 ± 1.98 mm, *p* = 0.001). The percentage of patients with diastolic dysfunction reduced from 43.0% before TAVI to 25.6% one month after the procedure (*p* < 0.001). 

Statistical analysis also showed an improvement in echocardiographic parameters used for PH assessment. Thus, the tricuspid peak gradient (37.57 ± 11.78 mmHg vs. 32.03 ± 9.01 mmHg, *p* < 0.001), the pulmonary artery acceleration time (PAAT, 91.49 ± 23.62 ms vs. 100.71 ± 21.56 ms, *p* < 0.001), the tricuspid annular plane systolic excursion (20.87 ± 4.26 mm vs. 21.47 ± 3.95 mm, *p* = 0.020) and the TAPSE/pulmonary arterial systolic pressure (PASP) ratio (0.45 ± 0.19 vs. 0.57 ± 0.20, *p* < 0.001) were statistically significantly improved parameters one month after TAVI. The severity of tricuspid regurgitation decreased secondary to TAVI, with a predominance of cases with minimal regurgitation (73.3% vs. 76.7%). The right pulmonary artery diameter (21.26 ± 2.52 mm vs. 20.52 ± 2.10 mm, *p* < 0.001) and inferior vena cava diameter (19.59 ± 2.94 mm vs. 18.67 ± 2.52 mm, *p* < 0.001) were also parameters with statistical value in the PH assessment before and one month after TAVI.

Considering the TAPSE/PASP ratio as an important predictor of PH, we divided the study group into two subgroups: group 1 with 45 patients (52.3%) with a ratio value less than 0.45 and group 2 with 41 patients (47.7%) with a ratio value above 0.45. Table 2 presents the average change (decrease or increase) of echocardiographic parameters used for PH and LV function assessment in the two groups mentioned above. The mean aortic gradient (*p* = 0.008), the aortic valve area (*p* = 0.013), LV ejection fraction (*p* = 0.049), PAAT value (*p* = 0.001), tricuspid peak gradient (*p* < 0.001) and improvement in the severity of tricuspid regurgitation (*p* = 0.016) are statistically significant parameters in assessing the evolution of patients undergoing TAVI in relation to the severity of PH.

**Figure 1:**
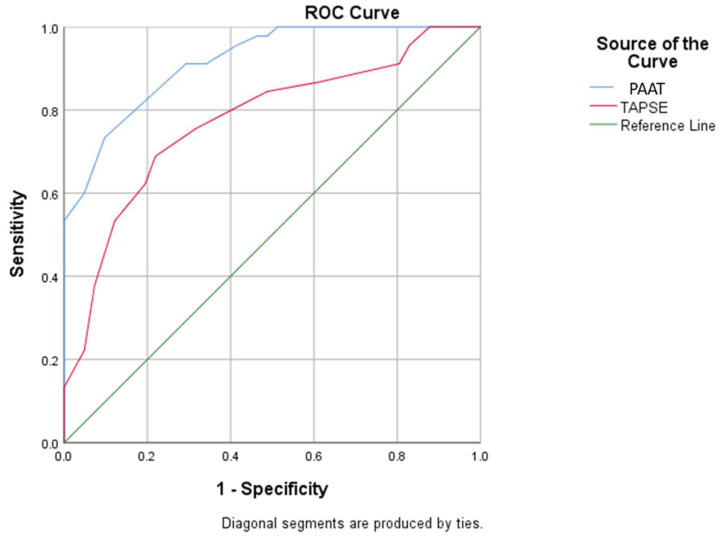
ROC curve using baseline PAAT and TAPSE values for prediction of severe pulmonary hypertension (PAAT: pulmonary artery acceleration time; TAPSE: tricuspid annular plane systolic excursion).

**Figure 2:**
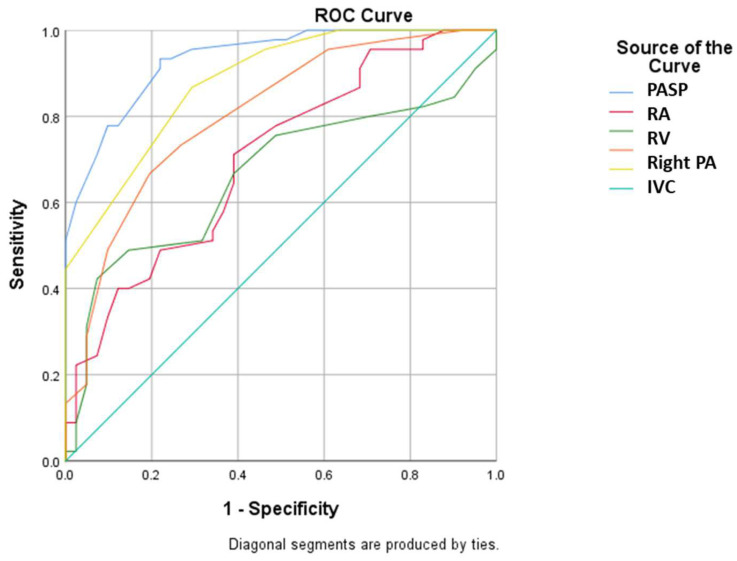
ROC curve using baseline PASP, RA, RV, right PA diameter and IVC values for prediction of severe pulmonary hypertension (PASP: pulmonary arterial systolic pressure; RA: right atrium; RV: right ventricle; PA: pulmonary artery; IVC: inferior vena cava).

Table 3 presents the predictive factors associated with a better prognosis one month after TAVI depending on the severity of preoperative PH. Statistical analysis showed a superior benefit following TAVI in patients with severe PH as assessed by the TAPSE/PASP ratio less than 0.45 (Figure 1 and Figure 2). ROC curve analysis established cut-off values for PAAT and TAPSE that allow the prediction of severe PH:

- A value less than 91.1 for PAAT has a sensitivity of 91.1% and specificity of 70.7% for predicting severe PH;
- A value less than 21.5 for TAPSE has a sensitivity of 75.6% and specificity of 68.3% for predicting severe PH (with the mention that establishing the diagnosis of severe PH requires evaluation and corroboration of several echocardiographic parameters).

## 4. Discussion

We evaluated a group of 86 patients diagnosed with severe aortic stenosis undergoing TAVI and demonstrated its beneficial role on both clinical (angina <*p* = 0.001> and fatigue <*p* < 0.001>) and echocardiographic parameters. Thus, we highlighted a number of predictors such as PAAT (*p* < 0.001), TAPSE (*p* = 0.020), PASP (*p* < 0.001) and the TAPSE/PASP ratio (*p* < 0.001) with a statistically significant role in assessing the severity of PH and thus prognosis.

Our study included predominantly female patients (53.3%), with a mean age of 75.8 ± 7.44 years old. The impact of demographics on the outcome in patients with severe AS undergoing TAVI has been investigated in various clinical trials to date. The role of gender differences in TAVI outcomes at 1 month has been less studied so far [26]. In a recent study, Zahid et al. [27] demonstrated that the risk of in-hospital death secondary to TAVI is higher in eighth decade patients due to the higher rate of vascular complications requiring blood transfusions [28]. Periprocedural risks have also been reported among men, who have a higher risk of acute kidney injury and need of pacemaker implantation. TAVI is predominantly performed in elderly patients who have an increased risk of adverse effects both associated with the previously administered bradycardia medication and the periprocedural risk of atrioventricular conduction disorders, which requires special consideration [29,30,31]. 

The aortic prostheses that were used in our study are also widely utilized worldwide, with numerous studies in the literature showing the clinical benefits of AS patients undergoing such a procedure [32]. Since the first TAVI procedure was performed in 2002 in a patient with AS, advances in technology and refinement of the method have led to the use of TAVI as an alternative to SAVR and, thus, to improved short- and medium-term prognosis in patients with severe aortic valve disease [33,34]. In our study, the Edwards Lifesciences Sapien 3 prosthesis was used in 74.4% of patients, with results obtained one month after evaluation demonstrating a clinical and functional improvement secondary to TAVI. The Placement of Aortic Transcatheter Valves (PARTNER) trial [35], patients who underwent TAVI were implanted with a Edwards SAPIEN valve prosthesis, with the one-year evaluation demonstrating both a 20% reduction in mortality and an 18.3% reduction in the associated risk of death or stroke [36]. Beneficial results were also reported at the 2-year follow-up, with the all-cause death rate being 43.3% lower compared to AS patients who received only medical therapy (*p* < 0.001). 

In a similar clinical trial, Reynolds et al. [37] applied the Kansas City Cardiomyopathy Questionnaire to a total of 628 patients with severe AS who were randomized into two distinct subgroups undergoing TAVI or SAVR and demonstrated an improvement in quality of life at both 1 month and 6 or 12 months for patients treated with TAVI (*p* < 0.001). Differences have also been reported in the type of approach used, with transfemoral TAVI being associated with improved prognosis compared to SAVR at 1 year after surgery. No clinical benefit was reported at 1 year after TAVI in patients in whom the transapical approach was chosen. 

In our study, both types of approaches were used, predominantly transfemoral (83.7% of cases), according to the experience of the interventional cardiologists in our center. The differences associated with the two types of vascular approaches were also investigated by Rougé, et al. [38] who observed a higher mortality rate for the apical approach compared to the transfemoral approach secondary to the prolonged learning curve and potential hemodynamic complications that may occur (*p* = 0.049). 

The investigators also identified a number of predictors of mortality, including female gender (*p* = 0.053), heart failure (class II-IV NYHA) (*p* = 0.09), concomitant coronary artery disease (*p* = 0.07) or apical approach (*p* = 0.08) [38].

The benefits of using the CoreValve Evolut R prosthesis were evaluated in the multicenter Medtronic CoreValve ADVANCE Study trial, in which a total of 1015 patients from 44 TAVI centers located in 12 different countries were included [39]. Patients showed a survival rate of 95.5% at 1 month and 87.2% at 6 months, supporting the use of TAVI in patients at high surgical risk. The investigators also reported a low complication rate, the main entities assessed being stroke (2.9% risk at 1 month) and acute vascular event risk (8.3% at 1 month).

Improvement in systolic and diastolic function of LV secondary to TAVI resulted in improved quality of life for patients by improving symptoms and New York Heart Association (NYHA) functional class of heart failure after one month. In our study, the percentage of patients with angina pectoris (*p* < 0.001) or fatigue (*p* < 0.001) was reduced by half after TAVI. The improvement of echocardiographic parameters also led to an improvement of the NYHA functional class, so that the severe impairment specific to class IV NYHA disappeared and the percentage of patients with symptoms specific to class III NYHA decreased from 72.1 to 18.6% at the one-month follow-up (*p* < 0.001). In a similar study, Adamo et al. [40] demonstrated that patients with an TAPSE/PASP ratio ≥0.36 compared to those with a ratio value below 0.36 mm/mmHg were associated with more comorbidities and were more symptomatic. Furthermore, patients in the second category with an X-ratio below 0.36 mm/mmHg with an associated increased risk of mortality after TAVI, being an independent parameter associated with surgical risk scores.

TAVI is associated with improvement in systolic and diastolic function after one month. In the literature there are studies showing the early effect of LV remodeling after TAVI compared to patients undergoing SAVR where regression of LV hypertrophy and normalization of diastolic dysfunction occurs late, over years [41,42,43]. The presence and progression of diastolic dysfunction in patients with severe AS increases the degree of fibrosis in the LV and thus negatively influences the patient’s prognosis in the short (one month after TAVI) or medium term [44,45]. 

Among the parameters with statistical significance in our study was the TAPSE/PASP ratio, an independent predictive factor in the assessment of severe PH (*p* < 0.001). Patients with a ratio value of less than 0.45 had an additional clinical benefit at the one-month evaluation after TAVI secondary to more pronounced improvements in echocardiographic parameters, indirectly leading to an increased quality of life and improved functional status.. The increase in aortic valve area, LV ejection fraction, decrease in tricuspid peak gradient or improvement in tricuspid regurgitation were significantly greater in these patients. The statistical significance of the TAPSE/PASP ratio in the assessment of severe PH was demonstrated by Tello et al. [25] in a study in which 52 patients with severe PH were included. This ratio also has value as an independent predictive factor in the assessment of pre-capillary PH as well as in the occurrence of heart failure, having a cut-off value of 0.36 mm/mmHg [46,47,48]. In a similar study, Uygur et al. [49] concluded that TAPSE/PASP ratio can be used as an independent predictor of long-term prognosis in terms of mortality or hospitalization rates. The presence of PH in patients with severe AS is frequent in these patients, but not quasi-prevalent, which raises the observation of its occurrence independently of the changes induced by valvulopathy [17,50]. In a similar clinical study, Sultan et al. [51] demonstrated the prognostic value of the TAPSE/PASP ratio in heart failure patients undergoing TAVI, its baseline value having an independent prognostic role in assessing the risk of all-cause death. Multivariate regression showed the persistence of statistical significance even after adjusting for confounders such as age, presence of atrial fibrillation, LV ejection fraction or cardiac output.

Both pre-capillary and post-capillary PH modulate the clinical outcome after TAVI [52]. O’Sullivan et al. [20] concluded that PH positively correlates with one-year mortality after TAVI (*p* = 0.004). Masri et al. [53] have shown that persistence of at least moderate PH at 1 month after TAVI is common and is associated with a higher risk of death from all causes. 

Drakopoulou et al. [54] observed a reduction in the mean PASP value after TAVI (*p* = 0.003) but with persistently increased mortality in patients with PH compared to those without (*p* < 0.001). Several clinical studies have shown a directly proportional relationship between the degree of PH severity and the 1-year mortality rate after TAVI [55]. In our study, in the first 30 days after TAVI, no patient died. Lindman et al. [56] demonstrated that gender differences and clinical factors influence mortality rate after TAVI more than hemodynamic parameters. In a similar clinical study, Barbash et al. [57] concluded in a similar study that a value of PASP over 50 mmHg is associated with an increased rate of death immediately after TAVI and a prolonged hospitalization. However, the data in the literature are inconclusive, as there are studies that attest to the lack of independent predictive value when identifying a PASP value above 60 mmHg [58]. 

The mean value of the PASP decreased post-TAVI at one month follow-up, thus being a statistically significant prognostic in our study (*p* < 0.001). Similar results have been reported in the literature, with persistence of elevated PASP values after TAVI being a marker associated with increased mortality [59]. A correlation between a decrease in PASP and an improvement in PH severity was also found, attesting to the prognostic benefit of TAVI, with recurrent PH usually being objectified 3–4 years after the procedure [60,61]. The importance of performing right heart catheterization prior to TAVI was highlighted by Maeder et al. [62] in a recent meta-analysis published in the literature, the association of invasive and echocardiographic data leading to improved diagnostic strategy in patients with severe AS. Increased pulmonary vascular resistance and the concomitant presence of pre- and post-capillary PH are predictive factors associated with a poor prognosis in patients with severe AS [62,63].

Not only PH, but also persistent tricuspid regurgitation is associated with a high risk of mortality after TAVI. In our study, the severity of tricuspid regurgitation (TR) improved at 30 days after TAVI, being more common among patients with a TAPSE/PASP ratio below 0.45 (*p* = 0.016). Yoshida et al. [64] investigated the outcome of persistent TR after TAVI and concluded that the presence of atrial fibrillation and a tricuspid annulus size greater than 37 mm are independent predictors associated with the persistence of TR after TAVI and thus increased all-cause mortality and hospitalizations due to heart failure. In a recent study, Olasińska-Wiśniewska et al. [65] demonstrated that the impact of PASP on long-term prognosis changes in patients undergoing TAVI. Thus, the assessment of PASP at one month after TAVI has a superior role in the assessment of the 1-year mortality rate compared to other parameters, with an increment of 1 mmHg increasing the mortality risk by 4%. Alushi et al. [66] evaluated PASP in 617 AS patients undergoing TAVI, demonstrating its reduction both at discharge and 1 year after surgery; patients with reversible PH after TAVI being associated with a low risk of death from any cause in the medium and long term. PASP also influences the short-term prognosis of patients after TAVI [67]. Patients with PASP over 49 mmHg had a longer duration of hospitalization, but no increased risk of readmission, stroke, invasive ventilatory support or bleeding episodes [17]. The effectiveness of SPAP in assessing mortality risk varies; however, with a number of clinical studies in the literature attesting to the independent prognostic role of persistence severe PH in assessing the risk of death after at least one year, but there is no correlation with a reduction in PH severity one month after TAVI [68].

Our study presents several limitations due to the small number of cases analyzed and the subjective assessment of associated symptoms. We excluded cases where the clinical-paraclinical data were incomplete as well as those patients who did not present for follow-up one month after TAVI. This was done to minimize the risk of misclassification, introducing a limited risk of selection bias. Furthermore, using a follow-up interval longer than 30 days may change the results of the statistical analysis and represents a future research direction for us.

## 5. Conclusions

In conclusion, we demonstrated in our study that PH influences TAVI results 30 days after the procedure, while identifying a number of predictors that indirectly assess the prognosis of patients with AS and severe PH. PAAT, TAPSE and PASP are the main statistically significant parameters that allow both the clinician and the cardiovascular surgeon to assess PH and indirectly the clinical and functional benefit of TAVI after only 30 days.

## Figures and Tables

**Table 1:** Demographics, resting hemodynamics, echocardiographic findings at baseline and at 1-month follow-up.

Parameters	Baseline	One Month Follow-Up	*p* Value
Age, y	75.8 ± 7.44		
Females	46 (53.3%)		
**Type of aortic prosthesis**			
Edwards Lifesciences Sapien 3	64 (74.4%)		
Medtronic Evolut R	22 (25.6%)		
**Vascular approach**			
Transfemoral	72 (83.7%)		
Transapical	14 (16.3%)		
**EUROSCORE II**	8.92 ± 10.60		
**Symptoms**			
Angina pectoris	63 (73.3%)	30 (34.9%)	<0.001
Fatigue	83 (96.5%)	41 (47.7%)	<0.001
**Comorbidities**			
Atrial fibrillation	21 (24.4%)	22 (25.6%)	1
Heart failure	NYHA class III 62 (72.1%)	NYHA class II 70 (81.4%)	<0.001
	NYHA class IV 24 (27.9%)	NYHA class III 16 (18.6%)	
**Resting hemodynamics**			
SBP, mmHg	149.07 ± 21.17	134.15 ± 11.21	<0.001
DBP, mmHg	82.72 ± 9.79	78.59 ± 8.64	<0.001
SaO_2_ (%)	89.76 ± 2.62	96.65 ± 1.59	<0.001
**Echocardiographic findings**			
Maximum aortic gradient (mmHg)	95.42 ± 24.86	24.06 ± 13.0	<0.001
Mean aortic gradient (mmHg)	59.22 ± 15.49	13.42 ± 9.83	<0.001
Aortic peak velocity (m/s)	4.79 ± 0.59	2.34 ± 0.51	<0.001
Aortic valve area (cm^2^)	0.69 ± 0.34	2.51 ± 0.39	<0.001
Aortic annulus size (mm)	22.47 ± 2.68	22.16 ± 2.82	0.046
Ascending aorta (mm)	33.27 ± 4.18	32.79 ± 4.34	0.086
LV ejection fraction (%)	54.45 ± 10.39	55.88 ± 9.45	0.007
LV end-diastolic diameter (mm)	49.34 ± 7.34	48.74 ±5.88	0.213
LV end-systolic diameter (mm)	34.85 ± 7.48	32.27 ± 7.17	0.019
Interventricular septum	14.42 ± 1.80	13.86 ± 1.98	0.001
Diastolic dysfunction	37 (43.0%)	22 (25.6%)	<0.001
LA diameter (mm)	58.74 ± 8.09	57.12 ± 8.01	0.012
Tricuspid peak gradient (mmHg)	37.57 ± 11.78	32.03 ± 9.01	<0.001
Tricuspid regurgitation			0.289
Absent	3 (3.5%)	4 (4.7%)	
Mild	63 (73.3%)	66 (76.7%)	
Moderate	16 (18.6%)	15 (17.4%)	
Moderate-severe	3 (3.5%)	1 (1.2%)	
Severe	1 (1.2%)	-	
PASP (mmHg)	47.80 ± 14.73	41.28 ± 11.18	<0.001
PAAT (ms)	91.49 ± 23.62	100.71 ± 21.56	<0.001
TAPSE (mm)	20.87 ± 4.26	21.47 ± 3.95	0.020
TAPSE/PASP	0.45 ± 0.19	0.57 ± 0.20	<0.001
RA diameter (mm)	48.37 ± 8.45	48.07 ± 7.61	0.410
RV diameter (mm)	32.37 ± 4.96	31.45 ± 4.21	0.050
Right PA diameter (mm)	21.26 ± 2.52	20.52 ± 2.10	<0.001
Inferior vena cava diameter (mm)	19.59 ± 2.94	18.67 ± 2.52	<0.001

All values are expressed as mean ± standard deviation (SD) or n (%); y: years; NYHA: New York Heart Association; SBP: systolic blood pressure; DBP: diastolic blood pressure; LV: left ventricle; LA: left atrium; PASP: pulmonary arterial systolic pressure; PAAT: pulmonary artery acceleration time; TAPSE: tricuspid annular plane systolic excursion; RA: right atrium; RV: right ventricle; PA: pulmonary artery.

**Table 2:** Evolution of echocardiographic parameters according to the TAPSE/PASP ratio value.

Parameter	Group 1 TAPSE/PASP ≤ 0.45	Group 2 TAPSE/PASP > 0.45	*p* Value
EUROSCORE II	−4.16 ± 11.41	−2.25 ± 5.86	0.090
Mean aortic gradient (mmHg)	−50.58 ± 16.1	−40.56 ± 18.04	0.008
Aortic peak velocity (m/s)	−2.57 ± 0.59	−2.29 ± 0.79	0.078
Aortic valve area (cm^2^)	1.98 ± 0.22	1.65 ± 0.71	0.013
LV ejection fraction (%)	2.42 ± 5.84	0.34 ± 2.85	0.049
PAAT (ms)	14.18 ± 14.37	3.78 ± 12.48	0.001
Tricuspid peak gradient (mmHg)	−8.91 ± 8.42	−1.83 ± 6.7	<0.001
Diastolic dysfunction	7 (15.6%)	9 (22.0%)	0.581
Tricuspid regurgitation	9 (20.0%)	1 (2.4%)	0.016

All values are expressed as mean ± standard deviation (SD) or n (%); LV: left ventricle; PAAT: pulmonary artery acceleration time.

**Table 3:** ROC curve using baseline echocardiographic parameters values for prediction of severe PH.

Parameter	Standard Error	*p* Value	AUC (95% C.I.)
PAAT	0.028	<0.001	0.916 (0.862–0.971)
TAPSE	0.051	<0.001	0.774 (0.674–0.873)
PASP	0.025	<0.001	0.934 (0.886–0.982)
RA diameter (mm)	0.056	0.001	0.702 (0.593–0.812)
RV diameter (mm)	0.663	0.009	0.663 (0.545–0.781)
Right PA diameter (mm)	0.047	<0.001	0.806 (0.714–0.898)
Inferior vena cava diameter (mm)	0.035	<0.001	0.880 (0.812–0.948)

AUC: area under the curve; C.I.: confidence interval; PASP: pulmonary arterial systolic pressure; PAAT: pulmonary artery acceleration time TAPSE: tricuspid annular plane systolic excursion; RA: right atrium; RV: right ventricle; PA: pulmonary artery.
